# Doxazosin oral intake therapy to relieve stent - related urinary symptoms and pain: a prospective, randomized, controlled study

**DOI:** 10.1590/S1677-5538.IBJU.2015.0570

**Published:** 2016

**Authors:** Long Zhang, Junping Li, Minjie Pan, Weiwei Han, Shucheng Liu, Yajun Xiao

**Affiliations:** 1 Department of Urology, Union Hospital, Tongji Medical College, Huazhong University of Science and Technology, Wuhan, Hubei, China; 2 Department of Urology, Jingmen No.1 People's Hospital, Jingmen, Hubei, China; 3 Department of Urology, Changzhou No.2 People's Hospital, Changzhou, Jiangsu, China

**Keywords:** Quality of Life, Doxazosin, Lower Urinary Tract Symptoms

## Abstract

**Objective:**

To assess the impact of Doxazosin Oral Intake Therapy on urinary symptoms and pain in patients with indwelling ureteral stents

**Patients and Methods:**

A total of 239 patients with ureteral stone-related hydronephrosis who underwent a double-J stent insertion after ureteroscopic lithotripsy were enrolled. Patients were randomized to receive doxazosin cotrolled release 4 mg once daily for 4 weeks or matching placebo. Patients completed the brief-form Chinese version Ureteric Stent Symptom Questionnaire (USSQ) and quality of life (QoL) score 2 weeks and 4 weeks after stent placement and 4 weeks after stent withdrawal. The analgesic use was also recorded during the stenting period.

**Results:**

Patients in Doxazosin Oral Intake Therapy group, in the first 2 weeks and second 2 weeks with the stent in situ, expressed significant lower daytime frequency (p=0.028 and p=0.038), nocturia (p=0.021 and p=0.008) and urgency (p=0.012 and p=0.014), respectively. Similarly, flank pain score, QoL score and analgesic use were also significant less in the stenting period. There was no significant difference in scores of urinary symptoms, pain and QoL during the post-stent period between two cohorts.

**Conclusions:**

Doxazosin Oral Intake Therapy reduced stent-related urinary symptoms, pain and the negative impact on QoL.

## INTRODUCTION

More than four decades have passed since the first description of an indwelling ureteral stent by Zimskind et al. (1). Placement of ureteral stent after ureteroscopic procedure remains common practice. However, morbidity associated with the stent has been well documented. In particular, general health and work performance were impacted by bothersome urinary symptoms in 80% cases and pain in 78% (2, 3).

Several studies have demonstrated α-blockers, such as alfuzosin and tamsulosin, improving stent-related symptoms (4-7). However, the results showed some fluctuations (7). Doxazosin Oral Intake Therapy has been shown to offer enhanced delivery rate, pharmacokinetic profile and compliance in patients with benign prostate hyperplasia (BPH) (8, 9). In addition, it might interact with α1-adrenergic receptor subtype located in central nervous system (CNS) for producing maximum benefit (9, 10). Herein, we firstly performed a double-blind, randomized, placebo controlled study to assess the impact of Doxazosin Oral Intake Therapy on stent-related urinary symptoms and pain.

## PATIENTS AND METHODS

This study was conducted with the institutional review approval. From June 2013 to February 2015, patients with unilateral hydronephrosis associated with ureteral stone to be treated with ureteroscopic lithotripsy and an indwelling ureteral stent insertion for 4 weeks were assumed for enrollment in the study. After documentation of informed consent, patients were randomized (using a Table with random number) to 1 of 2 treatment groups: doxazosin controlled release 4mg daily for 4 weeks (Carduran XL®, Pfizer Pharmaceuticals, Vega Baja) or matching placebo once daily for 4 weeks. Both investigators and patients were blinded to the randomization scheme and medication. Patients were asked to take 1 tablet on postoperative day 1.

Patients with bilateral stones, pregnancy or probable pregnancy, nursing, symptoms of BPH, urinary tract infection, chronic pain history, α-blocker or anticholinergic use in previous 3 months, hypotension or orthostatic hypotension history (resting blood pressure less than 100/70mmHg), age less than 18 years and other contraindications indicated in package insert were excluded.

Ureteroscopic lithotripsy was initially conducted with a Wolf 8F/9.8F semirigid ureteroscope in all patients. Stones were fragmented to less than 2mm using 0.8J 10Hz Holmium: YAG laser and a 200μm fiber. When flexible ureteroscope was required, a 12/14F dual lumen ureteral access sheath (Flexor® DL, Cook Urologic, Spencer, IN) was introduced over a working guidewire. A 6Fr indwelling double-J stent (Inlay optima®, Bard Medical, Convington, GA) was placed under fluoroscopic and cystoscopic control. Appropriate length was evaluated according to patient height. Stent position was examined radiographically.

The primary endpoints were the assessment of urinary symptoms and pain. The secondary endpoints included QoL, analgesics use and treatment emergent adverse event (TEAE). To assess urinary symptoms and pain associated with ureteral stent, patients were asked to complete the brief-form Chinese version USSQ 2 weeks and 4 weeks after stent placement and 4 weeks after stent withdrawal (11). The outcomes obtained at 4 weeks after stent withdrawal were proposed to be the baseline data without the impact of ureteral stone and related hydronephrosis (6). Patients received a prescription for diclofenac sodium 50mg×10 suppositories. A log sheet of analgesic use was kept during the stenting period.

The brief-form Chinese version USSQ, a simplified condition-specific questionnaire for evaluation of stent-related urinary symptoms and pain in the past 2 weeks, was adopted from the validated International Prostate Symptom Scale (IPSS) (11). The procedure of back translation was performed to verify the utility for covering the urinary symptoms section and pain section of the original USSQ initiated by Joshi et al. (11, 12). Accordingly, QoL of IPSS was also assessed in our study (13). The individual index has a score ranging from 0 to 5 (or QoL score of 6), with high scores corresponding to worse outcomes.

A sample size of 218 patients was calculated based on a power of 80% and a significant level of 0.05 to detect 30% difference in flank pain scores and 26% difference in frequency scores in the first 2 weeks with the stent in situ (11). Descriptive statistics included mean±SD for continuous variables, number and percentage for categorical variables. Student t test, chi-square test and non-parameter Wilcoxon test were used, as appropriate. Analyses were performed on full per-protocol set population as it was determined more appropriate to exclude those who failed to complete the study. A p value <0.05 was considered significant. Statistic analyses were conducted using computer software (Package for Social Science 19.0, SPSS Inc., Chicago, IL).

## RESULTS

A total of 239 patients were enrolled, 231 of whom were randomly assigned to either Doxazosin Oral Intake Therapy or placebo group. The total number of patients that completed the study at 2 weeks and 4 weeks after stent placement and 4 weeks after stent withdrawal were 219, 212 and 201, respectively. The main reasons for discontinuation were loss during follow-up, protocol deviation, withdrawal of consent and TEAE.

Patient demographics are listed in Table-1. Average patient age was 45.7 years (range 26 to 66), and the overall distribution of female and male was 29% and 71%, respectively. There was no significant difference in age, gender, stone size, stone location, height and length of stent between 2 cohorts.

**Table 1:** Patient characteristics.

Variables		Doxazosin	Placebo	P value
No. of Patients		112	107	NA
Age (y), Mean ± SD (Range)		44.7 ± 9.4 (26-64)	46.6 ±8.2 (30-66)	0.12*
Gender, n (%)				0.46†
	Female	35(31)	28 (26)	
	Male	77(69)	79(74)	
Laterality, n (%)				0.13†
	Left	53(47)	39(36)	
	Right	59(53)	68(64)	
Stone size (mm). Mean ±SD		8.2 ± 3.5	7.8 ±2.5	0.27*
Stone location, n (%)				0.56†
	Upper	19(17)	13 (12)	
	Middle	20(18)	18(17)	
	Lower	73(65)	76(71)	
Height (cm). Meant ± SD		164.4 ± 6.5	163.6 ± 6.2	0.31*
Length of stent, n (%)				0.35†
	24 cm	50(45)	55(51)	
	26 cm	62(55)	52 (49)	

NA not applicants

* Student t test

† Chi-square test

The overall study results are presented in Table-2. Patients receiving Doxazosin Oral Intake Therapy expressed significant lower daytime frequency (p=0.028 and p=0.038, respectively), nocturia (p=0.021 and p=0.008, respectively) and urgency (p=0.012 and p=0.014, respectively) in the first 2 weeks and second two weeks with the stent in situ when compared to the placebo group. Similarly, there were marked decreases in flank pain and abdominal pain that were associated with Doxazosin Oral Intake Therapy. Mean analgesics use and QoL scores were also significant lower in Doxazosin Oral Intake Therapy group during the stenting period.

**Table 2:** Overall study results.

Variables		Doxazosin	Placebo	P value
**Daytime Frequency**				
	First 2 weeks	2.18 ±1.80	2.68 ± 2.00	0.028
	Second 2 weeks	1.98 ±1.64	2.48 ±1.81	0.038
	Post-stent	1.08 ±1.24	1.33 ±1.41	0.266
**Nocturia**				
	First 2 weeks	1.35 ±1.46	1.88 ±1.74	0.021
	Second 2 weeks	1.05 ±1.18	1.66 ±1.62	0.008
	Post-stent	0.42 ± 0.83	0.49 ± 0.90	0.749
**Urgency**				
	First 2 weeks	1.33 ±1.67	1.93 ±1.74	0.012
	Second 2 weeks	1.13 ±1.33	1.67 ±1.57	0.014
	Post-stent	0.68 ±1.26	0.77 ± 0.97	0.083
**Urge incontinence**				
	First 2 weeks	0.09 ± 0.32	0.14 ±0.46	0.676
	Second 2 weeks	0.06 ± 0.23	0.08 ± 0.27	0.510
	Post-stent	0.00	0.02 ±0.14	0.232
**Hematuria**				
	First 2 weeks	1.20 ±1.62	1.26 ±1.52	0.644
	Second 2 weeks	0.86 ±1.28	1.05 ±1.25	0.141
	Post-stent	0.27 ± 0.56	0.16 ±0.43	0.196
**Flank pain**				
	First 2 weeks	1.13 ±1.46	1.83 ±1.92	0.007
	Second 2 weeks	0.93 ±1.18	1.45 ±1.54	0.014
	Post-stent	0.18 ±0.55	0.31 ± 0.67	0.140
**Abdominal pain**				
	First 2 weeks	1.03 ±1.30	1.60 ±1.74	0.015
	Second 2 weeks	0.80 ±1.08	1.50 ±1.55	0.001
	Post-stent	0.13 ±0.39	0.29 ± 0.66	0.074
**Urethral pain**				
	First 2 weeks	1.07 ±1.33	1.45 ±1.82	0.236
	Second 2 weeks	0.72 ±1.02	1.27 ±1.56	0.021
	Post-stent	0.18 ±0.54	0.21 ± 0.52	0.610
**QoL**				
	First 2 weeks	2.79 ±1.05	3.27 ±1.32	0.003
	Second 2 weeks	2.69 ± 0.97	3.00 ±1.07	0.024
	Post-stent	1.44 ±0.95	1.57 ±0.69	0.273
**Analgesics use**				
	First 2 weeks	0.30 ± 0.79	0.74 ±1.29	0.005
	Second 2 weeks	0.20 ± 0.62	0.53 ±1.02	0.006

Values in both treatment groups are presented by mean ± standard deviation

Mann-Whitney U test was used for all statistical analyses.

There was no significant difference in scores of urinary symptoms, pain and QoL in the post-stent period between two groups, which were considered as the baseline characteristics.

Four patients in Doxazosin Oral Intake Therapy experienced mild TEAE (dizziness in 3 and orthostatic hypotension in 1), and two of them discontinued medication, and symptoms disappeared after discontinuation.

## DISCUSSION

To our knowledge, this is the first double-blind, placebo controlled study to demonstrate the effectiveness of Doxazosin Oral Intake Therapy in reducing ureteral stent-related discomforts. A range of patient urinary symptoms and pain were improved, including frequency, nocturia, urgency, flank pain, abdominal pain. Patients QoL seemed to be better preserved also. The sample size per group in the study was the biggest in relevant literature. The same type of stent and indication were controlled to minimize related biases.

Although there have been lots of advances in ureteral stent composition and constructions design directed at improving biocompatibility and patient relevant symptoms, the ideal biomaterial for stent has yet to be discovered (14, 15). In contrary to stent engineering research, several studies suggested that α-blockers would be effective (4-7).

Deliveliotis et al. firstly reported that alfuzosin improved a subset of urinary symptoms and pain in patients with an indwelling double-J stent using USSQ 4 weeks after stent insertion (4). Then another two studies elucidating the efficacy of alfuzosin and tamsulosin on stent symptoms were performed by Buddingfield et al. and Damino et al., respectively (5, 6). Meta-analyses also showed that α-blockers are associated with improvements in stent symptoms (16, 17).

Recently, Dellis et al. reported that tamsulosin and alfuzosin reduced stent-related symptoms in patients with hydronephrosis associated ureteral stone who underwent stent placement, routinely for 4 weeks, after extracorporeal shock wave lithotripsy (SWL, n=106) or ureteroscopic lithotripsy (n=44) using the USSQ as originally intended by Joshi et al. 1 week and 4 weeks after stent placement and 4 weeks after stent removal (7). In our study, we assessed patient's morbidity in the first two week and second two weeks with the stent in situ and during the post-stent period using the brief-form Chinese version USSQ, which covers stent-related condition in recent two weeks. (11).

The pathophysiology of stent-related symptoms may be associated with irritation of the trigone, smooth muscle spasm in ureter and bladder, reflux of urine to kidney, or a combination of them (18). α1-adrenergic receptors are expressed in ureter, bladder body, bladder neck, urethra and prostate. The benefits of selective α1-blockers are 2-fold: inhibition of α1-adrenergic receptors in bladder neck and prostate to decrease outlet resistance and reflux of urine, and concurrent inhibition of α1-adrenergic receptors in bladder body and ureter to decrease bladder overactivity and ureter spasm, thereby improving both urinary symptoms and pain (19).

Doxazosin is a long-acting selective α1-adrenergic receptors antagonist with a half life of 16-20 hours. Doxazosin Oral Intake Therapy has been shown to offer enhanced pharmacokinetic profile, delivery rate and tolerability (8, 9). It may also improve lower urinary tract symptoms more completely via additional α-adrenergic receptors in CNS than tamsulosin (9, 10).

The beneficial effect of Doxazosin Oral Intake Therapy on stent symptoms was primarily shown in the first two weeks with the stent in situ. The results in the second two weeks with the stent in situ further indicated the efficacy of Doxazosin Oral Intake Therapy on stent symptoms, which were less dependent on patient surgical complications, preoperative condition and postoperative recovery. The outcomes obtained at 4 weeks after stent withdrawal were proposed to be the baseline data without the impact of ureteral calculi and related hydronephrosis (6). However, we still noted a trend for higher abdominal pain in the placebo group after stent removal; this finding might demonstrate the residual effects of the treatment or the prevalence of symptoms in background population (3).

Our study has several limitations. Firstly, we didn't evaluate all aspects of stent-related morbidity using USSQ initiated by Joshi et al., because the whole USSQ was not translated, adapted and validated in Chinese population. Secondly, Doxazosin Oral Intake Therapy was not compared with other α-blockers such as alfuzosin and tamsulosin. Thirdly, though the sample size per group was big enough for daytime frequency and flank pain, it might be relatively small to detect small differences of other parameters between two cohorts. Finally, since the brief-form Chinese version USSQ is used to assess symptoms associated with ureteral stent in previous 2 weeks, the discomforts were not evaluated on postoperative day 3 or 1 week after surgery when the outcomes were thought to be more relevant (5).

Future randomized study with larger patient population using validated USSQ is warranted to further ascertain the efficacy of Doxazosin Oral Intake Therapy on stent-related morbidity and find the best way to address those discomforts by comparison between Doxazosin Oral Intake Therapy and other drugs already studied, such as tamsulosin or anticholinergics.

## CONCLUSIONS

The administration of Doxazosin Oral Intake Therapy 4mg once daily reduced stent-related urinary symptoms, pain and the negative impact on QoL.

## Abbreviations

USSQ: = Ureteric Stent Symptom Questionnaire
QoL: = Quality of Life
TEAE: = Treatment Emergent Adverse Event
